# Polymorphisms of multiple genes involved in NER pathway predict prognosis of gastric cancer

**DOI:** 10.18632/oncotarget.10173

**Published:** 2016-06-20

**Authors:** Jingwei Liu, Na Deng, Qian Xu, Liping Sun, Huakang Tu, Zhenning Wang, Chengzhong Xing, Yuan Yuan

**Affiliations:** ^1^ Tumor Etiology and Screening Department of Cancer Institute and General Surgery, The First Affiliated Hospital of China Medical University, and Key Laboratory of Cancer Etiology and Prevention (China Medical University), Liaoning Provincial Education Department, Shenyang 110001, China

**Keywords:** nucleotide excision repair, gastric cancer, prognosis, polymorphism

## Abstract

Nucleotide excision repair (NER) is a versatile system that repairs various DNA damage. Polymorphisms of core NER genes could change NER ability and affect gastric cancer (GC) prognosis. We systematically analyzed the association between 43 SNPs of ten key NER pathway genes (*ERCC1*, *ERCC2*, *ERCC3*, *ERCC4*, *ERCC5*, *ERCC6*, *ERCC8*, *XPA*, *XPC*, and *DDB2*) and overall survival (OS) of 373 GC patients in Chinese. Genotyping was performed by Sequenom MassARRAY platform. We found for the first time that carriers of *ERCC2* rs50871 GG genotype demonstrated significantly increased hazards of death than GT/TT individuals (HR=2.55, *P*=0.002); *ERCC6* rs1917799 heterozygote GT were associated with significantly shorter OS than wild-type TT (adjusted HR=1.68, *P*=0.048); patients with *DDB2* rs3781619 GG genotype suffered higher hazards of death compared with AG/AA carriers (adjusted HR=2.30, *P*=0.003). Patients with *ERCC1* rs3212961 AA/AC genotype exhibited longer OS than CC genotype (adjusted HR=0.63, *P*=0.028); *ERCC5* rs2094258 AA/AG genotype revealed significantly favorable OS compared with GG genotype (adjusted HR=0.65, *P*=0.033); *DDB2* rs830083 CG genotype could increase OS compared with GG genotype (adjusted HR=0.61, *P*=0.042). Furthermore, patients simultaneously carrying two “hazard” genotypes exhibited even significantly worse survival with HR of 3.75, 3.76 and 6.30, respectively. Similarly, combination of “favorable” genotypes predicted better prognosis with HR of 0.56, 0.49 and 0.33, respectively. In conclusion, *ERCC2* rs50871 G/T, *ERCC6* rs1917799 G/T, *DDB2* rs3781619 A/G polymorphisms could predict shorter OS while *ERCC1* rs3212961 A/C, *ERCC5* rs2094258 A/G, *DDB2* rs830083 C/G polymorphisms could predict longer OS of GC, which might serve as promising biomarkers for GC prognosis.

## INTRODUCTION

Gastric cancer (GC) is the fourth most common cancer in the world and the second most common cause of cancer-related death [1]. Although remarkable improvement has been made in surgical treatment, the survival of GC still remains poor, with the overall 5-year survival rate for gastric cancer approximately 27.4% in China [2]. Moreover, patients with the same TNM (tumor/node/metastasis) stage and treatment may demonstrate various clinical outcomes. As a complex disease, the initiation and progression of GC is strongly influenced by both genetic and environmental factors [3, 4]. Therefore, identification of genetic biomarkers that could predict prognosis of GC patients would greatly benefit the individualized therapy, post-operational treatment and follow-up strategies [5].

DNA repair systems play a pivotal role in maintaining the stability and integrity of the genome, which include nucleotide excision repair (NER), base excision repair (BER), mismatch repair (MMR) and double-strand break repair (DSBR) [6, 7]. Nucleotide excision repair (NER) is a versatile system that monitors and repairs DNA damage caused by both endogenous and exogenous factors, including therapeutic agents [8]. As a result, the alternation of NER capacity could contribute to the different clinical outcomes of GC patients. NER process include steps of damage recognition, damage demarcation and unwinding, damage incision, and new strand ligation, all of which require corresponding functional proteins [9]. XPA, XPC and DDB2 are responsible for the DNA damage recognition [10, 11]; ERCC2 and ERCC3 participate in the damage unwinding process [12, 13]; ERCC1, ERCC4 and ERCC5 are involved in the DNA damage incision [14, 15]. In addition, ERCC6 and ERCC8 are both essential factors involved in transcription-coupled NER [16, 17].

Polymorphisms of core NER genes could change the NER ability by influencing the expression and function of important proteins, thereby altering individual survival of GC patients. Driven by such hypothesis, polymorphisms of several NER genes have previously been studied in relation to the prognosis of GC patients [18]. For example, Liu et al. studied *ERCC1* rs11615 C/T polymorphism and found that CT/TT genotype was significantly associated with worse prognosis compared with the CC genotype by evaluating overall survival (OS) in Chinese [19]. Han et al. investigated *ERCC1* rs3212986 A/C polymorphism in Korean population and revealed that GC patients with the CC genotype had longer OS than CA/AA carriers [20]. In addition, Zou et al. investigated the *ERCC5* rs17655 C/G polymorphism and found that GC patients with the CT/TT genotype had longer OS compared with CC genotype carriers in Chinese [21]. Although several NER polymorphisms have been reported to be related with survival of GC patients, most of these studies investigated only a few SNPs of a single NER gene. The association of NER gene polymorphisms with GC prognosis at the level of entire pathway and the joint effect of different polymorphisms of NER pathway genes remains largely unknown.

Until now, no study has yet been performed concerning the role of polymorphisms from perspective of the whole NER pathway in the prognosis of GC. In the present study, therefore, we systematically analyzed the association of 43 SNPs of ten key NER pathway genes (*ERCC1*, *ERCC2*, *ERCC3*, *ERCC4*, *ERCC5*, *ERCC6*, *ERCC8*, *XPA*, *XPC*, and *DDB2*) with survival of GC patients to investigate whether NER pathway polymorphisms could serve as potential biomarkers for GC prognosis.

## RESULTS

### Clinicopathological characteristics and OS of GC

A total of 373 patients including 263 (70.5%) males and 110 (29.5%) females were enrolled in the present study. The age of GC diagnosis ranged from 29 to 87 with a mean age of 58.8±10.2 years. At the last follow-up (September 2014), 114 patients died, with a median overall survival of 58.6 months. Among the patients, 184 (49.3%) presented with stages I-II and 189 (50.7%) with stages III-IV; 228 (61.1%) subjects were lymphatic metastasis positive while 145 (38.9%) patients were negative.

The effect of clinicopathological characteristics on survival of GC patients was summarized in Table 1. TNM stage (*P*<0.001), lymphatic metastasis (*P*<0.001), Borrmann classification (*P*=0.015) were all significant prognostic factors. No significant association was observed of Lauren's classification, alcohol drinking and smoking with GC survival. Therefore, multivariate analysis was subsequently performed using Cox's proportional hazards model adjusted by age, gender, TNM stage, lymphatic metastasis and Borrmann classification in order to identify independent prognostic value of polymorphism in NER pathway genes.

**Table 1:** Clinicopathological characteristics and OS of GC

Variables	Patients(%)	Deaths	MST(month)	HR(95%CI)	P
Age					
≦60	159(42.6)	65	60.6	1(Ref)	
>60	214(57.4)	49	52.5	1.08(0.74-1.56)	0.692
Gender					
Male	263(70.5)	80	58.7	1(Ref)	
Female	110(29.5)	34	44.8	0.95(0.64-1.42)	0.803
Growth pattern					
Expanding	27(9.2)	3	45.6	1(Ref)	
Intermediate	85(29.0)	17	41.7	2.38(0.70-8.15)	0.166
Infiltrative	181(61.8)	60	33.9	5.43(1.69-17.45)	0.004
Borrmann classification					
Borrmann I–II	82(24.5)	31	60.6	1(Ref)	
Borrmann III–IV	253(75.5)	81	48.4	1.36(1.09-2.08)	0.015
Lauren's classification					
Intestinal-type	136(36.9)	34	58.8	1(Ref)	
Diffuse-type	233(63.1)	78	56.6	0.70(0.46-1.04)	0.078
TNM stage					
I-II	184(49.3)	18	71.6	1(Ref)	
III-IV	189(50.7)	96	41.7	6.92(4.18-11.48)	<0.001
Lymphatic metastasis					
Negative	145(38.9)	16	70.6	1(Ref)	
Positive	228(61.1)	98	48.4	4.69(2.76-7.95)	<0.001
Alcohol drinking					
Nondrinkers	199(67.9)	55	37.5	1(Ref)	
Drinkers	94(32.1)	25	37.0	0.90(0.56-1.45)	0.665
Smoking					
Nonsmokers	183(62.5)	48	37.9	1(Ref)	
Smokers	110(37.5)	32	36.8	1.03(0.66-1.62)	0.894

### Polymorphisms of NER pathway genes and OS of GC

The results of the relation between all polymorphisms of NER pathway and GC survival in different genetic models were summarized in Supplementary Table S1. Of the 43 investigated SNPs in this study, six polymorphisms demonstrated significant association with GC survival, including *ERCC1* rs3212961 A/C, *ERCC2* rs50871 G/T, *ERCC5* rs2094258 A/G, *ERCC6* rs1917799 G/T, *DDB2* rs3781619 A/G and *DDB2* rs830083 C/G, which were shown in Table 2.

**Table 2:** NER pathway gene polymorphisms that demonstrate significant association with OS of GC

SNP	Compared Genotype	Patients(%)	Deaths	Crudea		Adjustedb	
				HR(95%CI)	P	HR(95%CI)	P
ERCC1 rs3212961	CC	92(25.9)	34	ref.		ref.	
	AC	189(53.2)	53	0.73(0.47-1.12)	0.149	0.67(0.43-1.04)	0.076
	AA	74(20.8)	17	**0.52(0.29-0.94)**	**0.031**	**0.54(0.30-0.99)**	**0.045**
	Dominant			0.66(0.44-1.00)	0.050	**0.63(0.41-0.95)**	**0.028**
ERCC2 rs50871	TT	125(35.2)	38	ref.		ref.	
	GT	207(58.3)	54	0.98(0.64-1.49)	0.912	0.89(0.58-1.36)	0.577
	GG	23(6.5)	12	**2.54(1.31-4.90)**	**0.006**	1.74(0.86-3.50)	0.121
	Recessive			**2.55(1.39-4.66)**	**0.002**	1.82(0.98-3.38)	0.059
ERCC5 rs2094258	GG	149(42.1)	53	ref.		ref.	
	AG	162(45.8)	39	**0.59(0.39-0.90)**	**0.014**	0.67(0.44-1.03)	0.068
	AA	43(12.1)	12	0.68(0.36-1.28)	0.231	0.69(0.36-1.32)	0.262
	Dominant			**0.61(0.42-0.90)**	**0.013**	**0.65(0.44-0.97)**	**0.033**
ERCC6 rs1917799	TT	70(32.6)	22	ref.		ref.	
	GT	105(48.8)	45	1.44(0.86-2.40)	0.162	**1.68(1.01-2.81)**	**0.048**
	GG	40(18.6)	15	1.23(0.64-2.37)	0.540	0.95(0.48-1.88)	0.874
	Dominant			1.40(0.85-2.26)	0.191	1.47(0.90-2.41)	0.124
DDB2 rs3781619	AA	132(37.2)	34	ref.		ref.	
	AG	187(52.7)	54	1.18(0.76-1.82)	0.456	1.20(0.77-1.88)	0.419
	GG	36(10.1)	16	**2.06(1.13-3.75)**	**0.018**	**2.40(1.27-4.55)**	**0.007**
	Recessive			**1.89(1.11-3.22)**	**0.019**	**2.30(1.33-3.97)**	**0.003**
DDB2 rs830083	GG	102(28.8)	32	ref.		ref.	
	CG	163(46.0)	45	0.66(0.41-1.04)	0.073	**0.61(0.38-0.98)**	**0.042**
	CC	89(25.1)	27	0.77(0.46-1.30)	0.324	0.81(0.48-1.38)	0.442
	Dominant			0.70(0.46-1.06)	0.093	0.66(0.43-1.01)	0.056

a Calculated by Cox proportional model using univariate analysis.

b Calculated by Cox proportional model using multivariate analysis.

**Figure 1:**
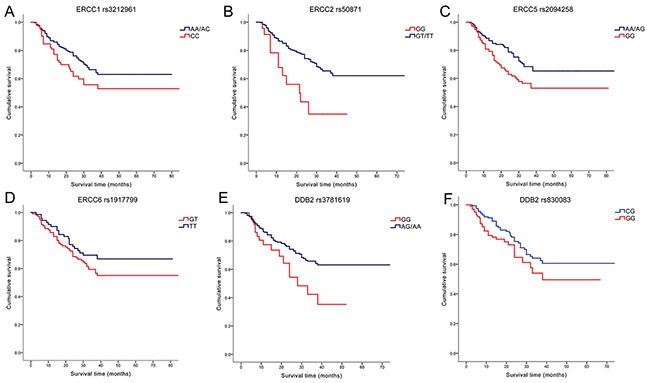
Kaplan–Meier survival curves by the genotypes of NER pathway polymorphisms in gastric cancer patients survival (**A.** *ERCC1* rs3212961 AA/AC vs. CC; **B.** *ERCC2* rs50871 GG vs. GT/TT; **C.** *ERCC5* rs2094258 AA/AG vs. GG; **D.** *ERCC6* rs1917799 GT vs. TT; **E.** *DDB2* rs3781619 GG vs. AG/AA; **F.** *DDB2* rs830083 CG vs. GG).

Carriers of *ERCC1* rs3212961AA genotype showed significantly favorable OS than wild-type CC genotype both in univariate and multivariate analysis (crude HR=0.52, 95%CI=0.29-0.94, *P*=0.031; adjusted HR=0.54, 95%CI=0.30-0.99, *P*=0.045); After adjustment, patients with the variant AA/AC genotype exhibited longer OS than with those who had CC genotype (HR=0.63, 95%CI=0.41-0.95, *P*=0.028) (Figure 1A). For *ERCC2* rs50871 G/T polymorphism, GG genotype subjects demonstrated significantly increased hazards of death in univariate model (GG vs. TT: HR=2.54, 95%CI=1.31-4.90, *P*=0.006; GG vs. (GT+TT): HR=2.55, 95%CI=1.39-4.66, *P*=0.002) (Figure 1B). *ERCC5* rs2094258 AG genotype patients were found to have longer OS than wild-type GG carriers in univariate model (HR=0.59, 95%CI=0.39-0.90, *P*=0.014); (AA+AG) genotype individuals revealed significantly favorable survival both in univariate and multivariate analysis compared with GG genotype (crude HR=0.61, 95%CI=0.42-0.90, *P*=0.013; adjusted HR=0.65, 95%CI=0.44-0.97, *P*=0.033) (Figure 1C). Patients of *ERCC6* rs1917799 heterozygote GT were associated with significantly shorter OS than TT genotype carriers after adjustments (adjusted HR=1.68, 95%CI=1.01-2.81, *P*=0.048) (Figure 1D). Patients with *DDB2* rs3781619 GG genotype suffered higher hazards of death with adjustment of confounding factors (GG vs. AA: HR=2.40, 95%CI=1.27-4.55, *P*=0.007; GG vs. (AG+AA): HR=2.30, 95%CI=1.33-3.97, *P*=0.003) (Figure 1E). For *DDB2* rs830083 C/G polymorphism, CG genotype could increase OS compared with GG genotype in multivariate model (HR=0.61, 95%CI=0.38-0.98, *P*=0.042) (Figure 1F).

To limit spurious findings, we attempted to use the Bonferroni correction for multiple comparisons considering significance thresholds for SNP association as *P*=1.16×10^−3^(0.05/43 SNPs). This is a fairly stringent correction given that not all of the SNPs analyzed were independent of each other because of linkage disequilibrium of SNPs. However, most results become not significant after Bonferroni correction.

### Polymorphisms of NER pathway genes and OS of GC in different subgroups

Stratification analysis was further performed to explore the relation of NER pathway gene polymorphisms and OS of GC in different subgroups. As was displayed in Table 3, the relation of *ERCC1* rs3212961 with favorable GC survival remained significant in subgroups of female, age≦60, Borrmann I–II, TNM stage III-IV, lymphatic metastasis and intestinal-type. *ERCC2* rs50871 had a better prediction value for worse GC prognosis in subgroups of female and age≦60 after adjustment. (AA+AG) genotype of *ERCC5* rs2094258 polymorphism could predict better OS in patients subgroups of age>60, Borrmann III–IV, TNM stage III-IV, lymphatic metastasis and diffuse-type. *ERCC6* rs1917799 GT genotype was associated with shorter survival in TNM stage III-IV and smokers. For *DDB2* rs3781619 polymorphism, significant relations were found in subgroups of age≦60, Borrmann III–IV, TNM stage III-IV, lymphatic metastasis and diffuse-type. *DDB2* rs830083 CG genotype could predict favorable survival in males, Borrmann III–IV, TNM stage III-IV, lymphatic metastasis and intestinal-type.

**Table 3:** Polymorphisms of NER pathway genes and OS of GC in different subgroups

Variables	Subgroup	Genotype (deaths/patients)		Crude^a^		Adjusted^b^	
				HR(95%CI)	P	HR(95%CI)	P
**ERCC1 rs3212961 (AA+AC) vs. CC**		**CC**	**AA+AC**				
Age	≦60	20/49	38/153	**0.54(0.31-0.93)**	**0.026**	**0.54(0.31-0.94)**	**0.028**
	>60	14/43	32/110	0.88(0.47-1.66)	0.700	0.77(0.41-1.46)	0.425
Gender	Male	22/64	54/189	0.77(0.47-1.27)	0.308	0.72(0.43-1.19)	0.199
	Female	12/28	16/74	**0.46(0.22-0.98)**	**0.043**	**0.45(0.21-0.97)**	**0.043**
Macroscopic type	Borrmann I–II	12/23	11/49	**0.33(0.15-0.75)**	**0.008**	**0.41(0.17-0.97)**	**0.043**
	Borrmann III–IV	21/58	58/189	0.82(0.50-1.36)	0.441	0.74(0.44-1.22)	0.236
TNM stage	I-II	5/46	11/130	0.77(0.27-2.22)	0.629	0.91(0.31-2.69)	0.863
	III-IV	29/46	59/133	**0.61(0.39-0.95)**	**0.028**	**0.59(0.37-0.93)**	**0.023**
Lymphatic metastasis	Negative	5/37	9/102	0.64(0.21-1.90)	0.416	0.82(0.25-2.74)	0.749
	Positive	29/55	61/161	**0.63(0.41-0.98)**	**0.042**	**0.62(0.39-0.97)**	**0.035**
Lauren's classification	Intestinal-type	13/37	19/93	0.55(0.27-1.11)	0.097	**0.45(0.22-0.95)**	**0.037**
	Diffuse-type	20/54	50/167	0.72(0.43-1.22)	0.223	0.71(0.42-1.21)	0.211
**ERCC2 rs50871 GG vs. (GT+TT)**		**GT+TT**	**GG**				
Age	≦60	49/184	9/18	**2.75(1.34-5.64)**	**0.006**	**2.16(1.04-4.47)**	**0.039**
	>60	43/148	3/5	2.20(0.68-7.13)	0.189	1.16(0.35-3.83)	0.805
Gender	Male	66/233	10/20	**2.22(1.14-4.33)**	**0.019**	1.46(0.74-2.86)	0.274
	Female	26/99	2/3	**8.27(1.84-37.12)**	**0.006**	**7.88(1.67-37.14)**	**0.009**
Macroscopic type	Borrmann I–II	20/68	3/4	**3.74(1.10-12.72)**	**0.035**	3.18(0.78-12.87)	0.106
	Borrmann III–IV	71/229	8/18	1.71(0.82-3.57)	0.149	1.43(0.68-3.01)	0.346
Lymphatic metastasis	Negative	13/131	1/8	2.45(0.32-19.07)	0.392	1.90(0.21-17.10)	0.567
	Positive	79/201	11/15	**2.26(1.20-4.26)**	**0.011**	1.78(0.93-3.40)	0.081
Lauren's classification	Intestinal-type	28/122	4/8	2.87(1.00-8.23)	0.050	1.47(0.48-4.49)	0.495
	Diffuse-type	62/206	8/15	**2.50(1.19-5.24)**	**0.015**	1.89(0.88-4.03)	0.102
**ERCC5 rs2094258 (AA+AG) vs. GG**		**GG**	**AA+AG**				
Age	≦60	27/79	31/122	0.63(0.37-1.05)	0.078	0.64(0.37-1.10)	0.106
	>60	26/70	20/83	0.61(0.34-1.09)	0.095	**0.50(0.28-0.92)**	**0.025**
Gender	Male	37/120	39/150	**0.63(0.40-0.98)**	**0.042**	0.63(0.40-1.00)	0.051
	Female	16/47	12/55	0.58(0.27-1.22)	0.149	0.57(0.26-1.25)	0.160
Macroscopic type	Borrmann I–II	10/28	13/44	0.79(0.35-1.80)	0.569	0.76(0.32-1.79)	0.528
	Borrmann III–IV	42/107	37/139	**0.58(0.37-0.91)**	**0.017**	**0.61(0.39-0.96)**	**0.034**
TNM stage	I-II	4/69	12/107	1.84(0.59-5.72)	0.289	1.91(0.60-6.02)	0.271
	III-IV	49/80	39/98	**0.54(0.35-0.82)**	**0.004**	**0.53(0.34-0.82)**	**0.004**
Lymphatic metastasis	Negative	6/60	8/79	0.89(0.31-2.56)	0.823	1.04(0.36-3.10)	0.951
	Positive	47/89	43/126	**0.55(0.36-0.83)**	**0.005**	**0.62(0.40-0.94)**	**0.025**
Lauren's classification	Intestinal-type	14/50	18/79	0.80(0.40-1.62)	0.540	1.03(0.50-2.12)	0.943
	Diffuse-type	39/97	31/124	**0.51(0.32-0.82)**	**0.006**	**0.51(0.31-0.83)**	**0.007**
**ERCC6 rs1917799 GT vs. TT**		**TT**	**GT**				
Macroscopic type	Borrmann I–II	11/26	13/32	1.03(0.46-2.29)	0.948	0.83(0.36-1.94)	0.669
	Borrmann III–IV	11/37	31/56	**2.03(1.02-4.04)**	**0.044**	**2.42(1.20-4.87)**	**0.014**
Smoking	Nonsmokers	7/30	16/41	1.73(0.71-4.21)	0.227	2.06(0.84-5.03)	0.114
	Smokers	4/18	14/32	2.11(0.69-6.41)	0.189	**4.10(1.19-14.06)**	**0.025**
**DDB2 rs3781619 GG vs. (AG+AA)**		**AG+AA**	**GG**				
Age	≦60	47/178	11/24	**2.16(1.12-4.19)**	**0.022**	**3.16(1.55-6.43)**	**0.001**
	>60	41/141	5/12	1.41(0.56-3.57)	0.469	1.45(0.56-3.77)	0.446
Macroscopic type	Borrmann I–II	19/65	4/7	2.12(0.72-6.23)	0.172	2.84(0.92-8.75)	0.069
	Borrmann III–IV	68/223	11/24	1.83(0.96-3.47)	0.065	**2.02(1.05-3.89)**	**0.035**
TNM stage	I-II	13/156	3/20	2.36(0.67-8.29)	0.181	2.60(0.66-10.20)	0.171
	III-IV	75/163	13/16	**2.08(1.15-3.76)**	**0.015**	**2.05(1.09-3.84)**	**0.026**
Lymphatic metastasis	Negative	11/119	3/20	2.09(0.58-7.50)	0.260	2.19(0.57-8.45)	0.254
	Positive	77/200	13/16	**2.66(1.47-4.79)**	**0.001**	**2.23(1.21-4.11)**	**0.010**
Lauren's classification	Intestinal-type	30/123	2/7	1.33(0.32-5.56)	0.700	3.45(0.74-16.12)	0.115
	Diffuse-type	56/192	14/29	**1.91(1.06-3.43)**	**0.032**	**2.03(1.10-3.75)**	**0.024**
**DDB2 rs830083 CG vs. GG**		**GG**	**CG**				
Gender	Male	30/75	30/121	**0.42(0.25-0.71)**	**0.001**	**0.45(0.26-0.76)**	**0.003**
	Female	2/27	15/42	**4.51(1.03-19.81)**	**0.046**	3.44(0.75-15.68)	0.111
Macroscopic type	Borrmann I–II	5/16	15/37	1.34(0.49-3.68)	0.573	1.07(0.38-3.03)	0.905
	Borrmann III–IV	25/78	30/111	**0.56(0.32-0.97)**	**0.038**	**0.56(0.33-0.98)**	**0.041**
TNM stage	I-II	3/54	8/78	1.03(0.27-3.94)	0.970	1.16(0.29-4.74)	0.834
	III-IV	29/48	37/85	**0.56(0.34-0.91)**	**0.019**	**0.58(0.35-0.97)**	**0.039**
Lymphatic metastasis	Negative	3/44	7/65	0.98(0.25-3.89)	0.981	0.85(0.20-3.60)	0.825
	Positive	29/58	38/98	**0.59(0.36-0.96)**	**0.032**	**0.60(0.36-0.99)**	**0.045**
Lauren's classification	Intestinal-type	9/35	16/65	0.54(0.23-1.26)	0.154	**0.30(0.12-0.79)**	**0.015**
	Diffuse-type	23/67	29/97	0.75(0.43-1.30)	0.306	0.81(0.46-1.43)	0.467

a Calculated by Cox proportional model using univariate analysis.

b Calculated by Cox proportional model using multivariate analysis.

### Polymorphisms of NER pathway genes and OS of GC in patients who received postoperative chemotherapy

We then performed survival analysis for patients who received postoperative chemotherapy to investigate whether chemotherapy had influence on the association between polymorphisms of NER pathway genes and OS of GC. Altogether 94 patients received chemotherapy after surgery. The results indicated that carriers of *ERCC2* rs50871GG genotype showed significantly unfavorable OS than (GT+TT) genotype both in univariate and multivariate analysis (crude HR=3.48, 95%CI=1.16-10.44, *P*=0.026; adjusted HR=5.36, 95%CI=1.69-17.03, *P*=0.004); Patients with *DDB2* rs3781619 GG genotype suffered higher hazards of death with adjustment (GG vs. AA: HR=10.30, 95%CI=1.11-95.80, *P*=0.040; GG vs. (AG+AA): HR=6.73, 95%CI=1.20-37.61, *P*=0.030) (Supplementary Table S2).

### Joint effect of NER pathway gene polymorphisms on OS of GC

**Figure 2:**
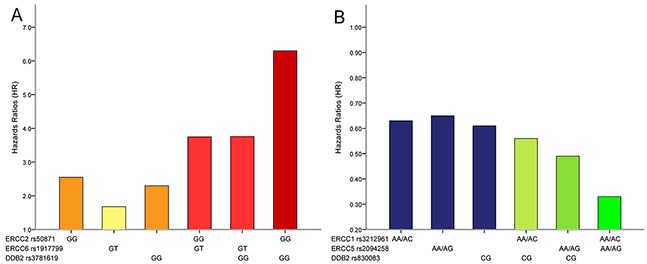
Combined detection of NER pathway polymorphisms could more effectively predict survival of gastric cancer patients (**A.** combined detection of polymorphisms that could predict worse survival; **B.** combined detection of polymorphisms that could predict favorable survival).

To investigate whether the combined detection of certain NER pathway polymorphisms could better predict the survival of GC, we further performed joint analysis of single NER polymorphism which demonstrated significant association (Table 4). The results indicated that patients simultaneously carrying two “hazard” genotypes exhibited even more significantly shorter OS (Figure 2A): carriers of both *ERCC2* rs50871 GG and *ERCC6* rs1917799 GT genotypes suffered a 3.75-fold increased hazards of death (*P*=0.019). Similarly, patients with both *ERCC2* rs50871 GG and *DDB2* rs3781619 GG genotypes (HR=6.30, *P*=0.001), with both *ERCC6* rs1917799 GT and *DDB2* rs3781619 GG genotypes (HR=3.76, *P*=0.006) showed significant worse survival.

**Table 4:** Joint effect of NER pathway gene polymorphisms on OS of GC

Combined Genotype		Patients(%)	Deaths	Crude^a^		Adjusted^b^	
				HR(95%CI)	P	HR(95%CI)	P
**ERCC2 rs50871**	**ERCC6 rs1917799**						
GT+TT	TT	61(37.7)	18	ref.		ref.	
GG	TT	4(2.5)	2	1.79(0.41-7.75)	0.439	0.98(0.21-4.65)	0.979
GT+TT	GT	90(55.6)	36	1.38(0.79-2.44)	0.261	1.57(0.89-2.78)	0.120
GG	GT	7(4.3)	5	**4.00(1.47-10.90)**	**0.007**	**3.75(1.24-11.32)**	**0.019**
**ERCC2 rs50871**	**DDB2 rs3781619**						
GT+TT	AA+AG	301(84.8)	80	ref.		ref.	
GG	AA+AG	18(5.1)	8	2.03(0.98-4.21)	0.056	1.54(0.73-3.25)	0.256
GT+TT	GG	31(8.7)	12	1.60(0.87-2.93)	0.132	**1.97(1.06-3.67)**	**0.032**
GG	GG	5(1.4)	4	**7.16(2.57-19.93)**	**1.65×10^−4^**	**6.30(2.19-18.18)**	**0.001**
**ERCC6 rs1917799**	**DDB2 rs3781619**						
TT	AA+AG	58(35.8)	16	ref.		ref.	
GT	AA+AG	87(53.7)	34	1.47(0.81-2.66)	0.204	1.70(0.94-3.10)	0.082
TT	GG	7(4.3)	4	2.33(0.78-6.99)	0.131	1.95(0.58-6.61)	0.282
GT	GG	10(6.2)	7	**3.16(1.30-7.69)**	**0.011**	**3.76(1.47-9.63)**	**0.006**
**ERCC1 rs3212961**	**ERCC5 rs2094258**						
CC	GG	39(11.0)	16	ref.		ref.	
AA+AC	GG	110(31.1)	37	0.70(0.39-1.26)	0.234	0.58(0.30-1.11)	0.099
CC	AA+AG	53(15.0)	18	0.68(0.35-1.35)	0.272	0.69(0.32-1.48)	0.343
AA+AC	AA+AG	152(42.9)	33	**0.42(0.23-0.76)**	**0.004**	**0.33(0.17-0.63)**	**0.001**
**ERCC1 rs3212961**	**DDB2 rs830083**						
CC	GG	27(10.2)	10	ref.		ref.	
AA+AC	GG	75(28.3)	22	0.78(0.37-1.65)	0.516	0.89(0.40-1.99)	0.779
CC	CG	49(18.5)	16	0.68(0.31-1.51)	0.343	0.69(0.29-1.66)	0.407
AA+AC	CG	114(43.0)	29	0.51(0.25-1.06)	0.070	0.56(0.26-1.20)	0.135
**ERCC5 rs2094258**	**DDB2 rs830083**						
GG	GG	41(15.5)	14	ref.		ref.	
AA+AG	GG	60(22.7)	18	0.88(0.44-1.78)	0.727	0.88(0.42-1.86)	0.733
GG	CG	66(25.0)	22	0.83(0.41-1.63)	0.589	0.79(0.37-1.67)	0.537
AA+AG	CG	97(36.7)	23	**0.49(0.25-0.96)**	**0.038**	**0.49(0.24-1.00)**	**0.048**

a Calculated by Cox proportional model using univariate analysis.

b Calculated by Cox proportional model using multivariate analysis.

The combination of single NER polymorphism predicting better prognosis also revealed even more favorable survival (Figure 2B): *ERCC1* rs3212961 AA/AC and *ERCC5* rs2094258 AA/AG genotypes carriers had a significant longer OS (HR=0.33, 95%CI=0.17-0.63, *P*=0.001); individuals with both *ERCC5* rs2094258 AA/AG and *DDB2* rs830083 CG genotypes were associated with significantly increased OS (HR=0.49, 95%CI=0.24-1.00, *P*=0.048). It was therefore obvious that combined detection of two core NER pathway gene polymorphisms could more effectively predict GC survival.

## DISCUSSION

Identifying biomarkers associated with GC survival is essential for the individualized therapy and post-operational treatment for different patients. Although several previous studies have revealed that NER gene polymorphisms could alter GC survival, to the best of our knowledge this is the first comprehensive investigation of the relationship between polymorphisms of the entire NER pathway and prognosis of GC. In this study, 43 SNPs of ten NER pathway genes were investigated in relation to OS of GC patients. We for the first time found that *ERCC2* rs50871 G/T, *ERCC6* rs1917799 G/T, *DDB2* rs3781619 A/G polymorphisms were significantly associated with shorter OS while *ERCC1* rs3212961 A/C, *ERCC5* rs2094258 A/G, *DDB2* rs830083 C/G could predict favorable OS of GC patients in Chinese. In addition, the combined detection of NER pathway gene polymorphisms could more effectively predict the prognosis of GC.

NER process includes steps of damage recognition, damage demarcation and unwinding, damage incision, and new strand ligation [22, 23]. Multiple genes are involved in this pathway and in charge of different functions [24]: XPA, XPC and DDB2 are responsible for the DNA damage recognition; ERCC2 and ERCC3 participate in the damage unwinding process; ERCC1, ERCC4 and ERCC5 are involved in the DNA damage incision; ERCC6 and ERCC8 are both essential factors involved in transcription-coupled NER. In this study, we found that *ERCC2* rs50871 GG, *ERCC6* rs1917799 GT and *DDB2* rs3781619 GG genotypes indicated worse OS of GC. The results for *ERCC2* rs50871 was opposite to the previous findings in melanoma [25] or head and neck cancer [26], in which the GG genotype was related to favorable survival. *ERCC6* rs1917799 was previously linked to increased risk of GC [27], but its role in GC prognosis has not been studied before. In this study, *ERCC6* rs1917799 heterozygote GT was firstly found to be associated with significantly shorter OS than wild-type TT. In addition, we found that *ERCC1* rs3212961 AA/AC, *ERCC5* rs2094258 AA/AG and *DDB2* rs830083 CG genotypes could predict favorable OS. After analyzing patients who received postoperative chemotherapy, we found that *ERCC2* rs50871 and *DDB2* rs3781619 polymorphisms were significantly associated with worse OS. However, due to the limited number of patients who received chemotherapy, the detailed role of chemotherapy in the relation between NER pathway polymorphisms and GC prognosis stilled need further investigations.

Among these newly discovered polymorphisms which could predict GC survival, *ERCC5* rs2094258 and *ERCC6* rs1917799 were both located in the 5′ upstream regulatory region, which may influence the binding activity of certain transcriptional factor, thus altering the expression and function of corresponding NER factors. *ERCC1* rs3212961, *ERCC2* rs50871, *DDB2* rs3781619 and *DDB2* rs830083 were all located in the intron region of genes. Sequence variation of introns especially the polymorphic site which changes alternative splicing patterns hold great promise in altering the regulation of the gene's transcription and thereby modulating function of specific factors of NER pathway [28]. By this way, these polymorphisms of NER pathway genes may modulate the DRC phenotype. The alteration in the NER capacity may in turn change frequencies of DNA mutation due to unrepaired damaged DNA. Thus, it is biologically plausible that polymorphisms in NER genes may influence clinical outcomes in GC patients. Furthermore, it is notable that the polymorphisms which demonstrated significant associations with GC survival are located within different genes responsible for each step of NER process: *DDB2* of “damage recognition” step, *ERCC2* of “damage unwinding” step, *ERCC1* and *ERCC5* of “damage incision” step. This interesting phenomenon suggested that each step of the NER process was important for the role of NER in GC prognosis. Although the above-mentioned mechanisms might, at least in part, explained the observed significant associations of certain NER polymorphisms and GC survival, further molecular researches are still needed to reveal the underlying mechanism.

As a complicated and multi-step process, NER pathway factors might function jointly to alter GC prognosis, and a single polymorphism may be insufficient to predict GC survival. Therefore, on the basis of our findings of certain relations between single polymorphism and GC survival, we further explored whether the combined detection of certain NER pathway gene polymorphisms could better predict the survival of GC through joint analysis of single NER polymorphism which demonstrated significant association with GC survival. The findings suggested that patients simultaneously carrying two “hazard” genotypes exhibited even more significantly worse OS: carriers of both *ERCC2* rs50871 GG and *ERCC6* rs1917799 GT genotypes suffered a 3.75-fold increased hazards of death; patients with both *ERCC2* rs50871 GG and *DDB2* rs3781619 GG genotypes (HR=6.30), with both *ERCC6* rs1917799 GT and *DDB2* rs3781619 GG genotypes (HR=3.76) showed significant worse OS. Similarly, the combination of single NER polymorphism predicting better prognosis also revealed even more favorable survival: *ERCC1* rs3212961 AA/AC and *ERCC5* rs2094258 AA/AG genotypes carriers had a significant longer survival (HR=0.33); individuals with both *ERCC5* rs2094258 AA/AG and *DDB2* rs830083 CG genotypes were associated with significantly increased OS (HR=0.49). Therefore, it was obvious that combined detection of two core NER pathway polymorphisms could more effectively predict GC survival. These results might due to the interaction or joint effect of polymorphisms of different factors involved in NER pathway. It is promising that the joint detection of different polymorphisms of NER pathway could be applied in the prediction of the prognosis of GC.

Several limitations should be acknowledged in this study. Considering the availability of our data, we could only get the information that whether the patients received chemotherapy after surgery rather than detailed chemotherapeutic information. Secondly, the sample size of this study was relatively insufficient especially for the subgroup analysis, which requires future studies based on large population to confirm. Thirdly, most results became not significant after Bonferroni correction which required stricter significance. Bonferroni correction might be a fairly stringent correction given that not all of the SNPs analyzed were independent of each other because of linkage disequilibrium of SNPs. We therefore considered the results of this study as preliminary screening and exploration, which provide direction for future studies concerning NER pathway and GC prognosis.

Our study for the first time unravelled the promising role of NER pathway gene polymorphism as a prognosis biomarker from the perspective of the entire pathway. Such NER pathway gene polymorphisms would largely benefit the management strategy for GC patients, making it possible to enhance the follow-up and dynamic monitoring for GC individuals with the specific genotype of certain polymorphisms.

In summary, our findings demonstrated that *ERCC2* rs50871 G/T, *ERCC6* rs1917799 G/T, *DDB2* rs3781619 A/G polymorphisms were significantly associated with shorter OS of GC; *ERCC1* rs3212961 A/C, *ERCC5* rs2094258 A/G, *DDB2* rs830083 C/G could predict favorable OS of GC patients in Chinese. Joint detection of *ERCC2* rs50871, *ERCC6* rs1917799 and *DDB2* rs3781619 could more efficiently predict worse OS while combined detection of *ERCC1* rs3212961, *ERCC5* rs2094258 and *DDB2* rs830083 could predict even better OS. Therefore, polymorphisms of multiple genes involved in NER pathway might serve as promising biomarkers to predict prognosis of gastric cancer.

## MATERIALS AND METHODS

### Study subjects

This study project was approved by the Institute Research Medical Ethics Committee of the First Affiliated Hospital of China Medical University. A total of 373 GC patients were recruited from the Department of Surgical Oncology of the First Affiliated Hospital of China Medical University between 2008 and 2013. Written informed consents were obtained from participants. Medical histories were acquired by questionnaire and the records were computerized. All the GC patients were histopathologically confirmed and classified based on current Borrmann and Lauren's classification. Tumors were staged using the 7th edition of the TNM staging system of the International Union Against Cancer (UICC)/American Joint Committee on Cancer (AJCC) (2010) according to postoperative pathologic examination. Patients who (i) had other malignant tumours (ii) distant metastasis found preoperatively (iii) underwent preoperative radiotherapy or chemotherapy were excluded from this study. And the prognostic parameter is overall survival (OS) in this study. OS was calculated up to either the date of death or last clinical follow-up, whichever occurred first. Patients without death at the time of the analysis were censored at the date of the last follow-up. The follow-up of the patients was completed by September 2014.

### Candidate genes and SNP selection

Genotype data from extended NER pathway gene regions encompassing 5 kb of upstream and downstream flanking sequences were extracted from the HapMap Chinese Han Beijing population (Release 27, Phase I + II + III, http://www.HapMap.org). Haploview software (http://www.broadinstitute.org/mpg/haploview) was used to minimize the number of SNPs needed to be genotyped, providing a significant shortcut to carry out candidate gene association studies in a particular population. Tag SNPs were selected on the basis of pairwise linkage disequilibrium information to maximally capture (r^2^ > 0.8) common or rare variants (minor allele frequency [MAF] > 0.05) by Haploview 4.2. FastSNP Search was used to predict the potential SNP function (leading to amino acid substitutions, altering splicing or transcription factor-binding motifs, acting as intronic enhancers) [29, 30]. Totally 43 SNPs covering ten key NER pathway genes (*ERCC1*, *ERCC2*, *ERCC3*, *ERCC4*, *ERCC5*, *ERCC6*, *ERCC8*, *XPA*, *XPC*, and *DDB2*) were eventually chosen by integrating these two publicly available tools. The detailed information of selected SNPs from NER pathway genes was shown in Table 5.

**Table 5:** Detailed information of 43 genotyped SNPs in NER pathway

Gene	dbSNP number	Base change	SNP location	MAF	
				In database	GC patients
ERCC1	rs11615	C>T	Exon	0.243	0.241
	rs2298881	C>A	Promoter	0.444	0.399
	rs3212955	A>G	Intron	0.289	0.300
	rs3212961	C>A	Intron	0.453	0.475
	rs3212986	G>T	3′ Untranslated region	0.310	0.330
	rs735482	A>C	3′ Untranslated region	0.427	0.435
ERCC2	rs1052555	C>T	Exon	0.104	0.068
	rs13181	T>G	Exon	0.095	0.082
	rs238406	G>T	Exon	0.407	0.461
	rs238417	G>C	Intron	0.488	0.461
	rs50871	T>G	Intron	0.279	0.356
	rs50872	C>T	Intron	0.190	0.221
ERCC3	rs4150441	G>A	Intron	0.444	0.424
	rs4150448	G>A	Intron	0.109	0.110
	rs4150506	C>T	Intron	0.320	0.307
ERCC4	rs6498486	A>C	5′ Upstream	0.282	0.225
	rs1799801	T>C	Exon	0.237	0.210
	rs2276464	G>C	3′ Untranslated region	0.275	0.207
	rs254942	T>C	Intron	0.241	0.215
ERCC5	rs1047768	T>C	Exon	0.241	0.312
	rs2094258	G>A	Promoter	0.383	0.350
	rs2228959	C>A	Exon	0.062	0.046
	rs2296147	T>C	5′ Upstream	0.201	0.218
	rs4150291	A>T	Intron	0.081	0.100
	rs4150383	G>A	Intron	0.088	0.063
	rs751402	C>T	Promoter	0.367	0.359
	rs873601	G>A	3′ Untranslated region	0.496	0.479
ERCC6	rs1917799	T>G	5′ Upstream	0.303	0.430
ERCC8	rs158572	A>G	Intron	0.306	0.107
	rs158916	T>C	5′ Upstream	0.152	0.121
XPA	rs10817938	T>C	5′ Upstream	0.208	0.236
	rs2808668	T>C	Intron	0.478	0.499
	rs3176629	C>T	Promoter	0.088	0.096
	rs3176658	C>T	Intron	0.256	/
XPC	rs1870134	G>C	5′ Upstream	0.244	0.269
	rs2228000	C>T	Exon	0.325	0.312
	rs2228001	A>C	Exon	0.372	0.375
	rs2470352	A>T	3′ UTR	0.058	0.007
	rs2607775	C>G	5′ Upstream	0.089	0.049
DDB2	rs2029298	G>A	Promoter	0.354	0.324
	rs326222	T>C	Intron	0.274	0.268
	rs3781619	A>G	Intron	0.383	0.365
	rs830083	C>G	Intron	0.367	0.482

MAF for Chinese in Hapmap database (www.hapmap.org)

Abbreviations: MAF, minor allele frequency; GC, gastric cancer.

### Genotyping assay

Genomic DNA was isolated from blood samples by routine phenol–chloroform extraction and then diluted into working concentrations (50 ng/μl) for further genotyping. Samples were placed randomly on the 384-well plates and blinded for the status of disease. The design of the assay and SNP genotyping were performed by Bio Miao Biological Technology (Beijing, China) using the Sequenom MassARRAY platform (Sequenom, San Diego, CA) according to the manufacturer's instructions. The results of all duplicated samples were 100% consistent.

### Statistical analysis

Statistical analysis was performed by using SPSS (16.0) statistical software (SPSS, Chicago, IL, USA). The Kaplan-Meier method was applied to visualize overall survival (OS) by different genotype groups. The median survival time (MST) was calculated; mean survival time was chosen if the median survival time could not be calculated. The log-rank test was used to test for equality of the survival distributions. Univariate and multivariate Cox proportional hazards models were performed to calculate crude or adjusted hazards ratios (HR) and 95% confidence intervals (CI) of each genotype to estimate its effect on OS with or without adjustment for confounding factors. Significant variables in univariate models were further analyzed by multivariate Cox proportional hazards regression models to identify the independent prognostic value. Two-tailed *P* values < 0.05 were considered statistically significant.

## SUPPLEMENTARY TABLES




