# Supplementary material for: Polymorphisms of multiple genes involved in NER pathway predict prognosis of gastric cancer
Source: Oncotarget. 2016 Jun 20;7(30):48130–42. doi: 10.18632/oncotarget.10173 (PMC5217006; doi:10.18632/oncotarget.10173)
Supplement: Supplementary file 1 [file oncotarget-07-48130-s001.pdf]

Polymorphisms of multiple genes involved in NER pathway predict prognosis of gastric cancer

SUPPLEMENTARY TABLES

Supplementary Table S1: Associations between polymorphisms of NER pathway genes and gastric cancer survival in different genetic models.

See Supplementary File 1

Supplementary Table S2: Polymorphisms of NER pathway genes and OS of GC in patients who received postoperative chemotherapy

SNP	Compared Genotype	Patients(%)	Deaths	Crude[a]		Adjusted[b]	
				HR(95%CI)	P	HR(95%CI)	P
ERCC1 rs3212961	CC	23(24.2)	7	ref.		ref.	
	AC	51(53.7)	9	0.64(0.24-1.72)	0.374	0.72(0.26-1.99)	0.528
	AA	18(18.9)	6	1.28(0.43-3.80)	0.662	2.71(0.79-9.30)	0.114
	Dominant			0.79(0.32-1.94)	0.607	0.96(0.39-2.40)	0.934
ERCC2 rs50871	TT	28(29.5)	7	ref.		ref.	
	GT	57(60.0)	11	0.75(0.29-1.95)	0.550	0.58(0.21-1.60)	0.290
	GG	7(7.4)	4	2.82(0.78-10.15)	0.112	3.24(0.84-12.5)	0.088
	Recessive			**3.48(1.16-10.44)**	**0.026**	**5.36(1.69-17.03)**	**0.004**
ERCC5 rs2094258	GG	39(41.1)	12	ref.		ref.	
	AG	39(41.1)	7	0.49(0.19-1.25)	0.137	0.38(0.14-1.03)	0.057
	AA	13(13.7)	3	0.67(0.19-2.37)	0.531	0.57(0.14-2.38)	0.441
	Dominant			0.54(0.23-1.24)	0.145	0.42(0.18-1.03)	0.057
ERCC6 rs1917799	TT	15(15.8)	4	ref.		ref.	
	GT	15(15.8)	3	0.72(0.16-3.24)	0.673	1.03(0.18-5.83)	0.970
	GG	6(6.3)	3	1.88(0.42-8.40)	0.410	1.50(0.25-9.10)	0.659
	Dominant			1.05(0.30-3.73)	0.939	0.96(0.26-3.54)	0.956
DDB2 rs3781619	AA	37(38.9)	8	ref.		ref.	
	AG	49(51.6)	12	1.23(0.50-3.01)	0.654	1.20(0.48-3.03)	0.694
	GG	6(6.3)	2	2.11(0.44-10.21)	0.353	**10.30(1.11-95.80)**	**0.040**
	Recessive			1.86(0.43-8.03)	0.405	**6.73(1.20-37.61)**	**0.030**
DDB2 rs830083	GG	27(28.4)	6	ref.		ref.	
	CG	43(45.3)	11	0.83(0.29-2.32)	0.714	0.84(0.28-2.50)	0.757
	CC	21(22.1)	5	1.08(0.33-3.54)	0.898	1.10(0.32-3.76)	0.878
	Dominant			0.90(0.34-2.35)	0.828	0.86(0.32-2.31)	0.771

[a], Calculated by Cox proportional model using univariate analysis.

[b], Calculated by Cox proportional model using multivariate analysis.
