# Supplementary material for: Polymorphisms of multiple genes involved in NER pathway predict prognosis of gastric cancer
Source: Oncotarget. 2016 Jun 20;7(30):48130–42. doi: 10.18632/oncotarget.10173 (PMC5217006; doi:10.18632/oncotarget.10173)
Supplement: Supplementary file 2 [file oncotarget-07-48130-s002.doc]

Supplementary Table 1. Associations between polymorphisms of NER pathway genes and gastric cancer survival in different genetic models.

|  |  |  |  |  |  | **Crude^a^** | |  | **Adjusted^b^** | |
| --- | --- | --- | --- | --- | --- | --- | --- | --- | --- | --- |
| **Gene** | **SNP** | **Genotype** | **Patients(%)** | **Death** |  | **HR(95%CI)** | **P** |  | **HR(95%CI)** | **P** |
| ERCC1 | rs11615 | CC | 202(56.9) | 55 |  | ref. |  |  | ref. |  |
|  |  | CT | 135(38.0) | 43 |  | 1.36(0.91-2.03) | 0.136 |  | 1.25(0.83-1.86) | 0.285 |
|  |  | TT | 18(5.1) | 6 |  | 1.14(0.49-2.65) | 0.760 |  | 1.03(0.40-2.61) | 0.957 |
|  |  | Dominant |  |  |  | 1.33(0.90-1.96) | 0.147 |  | 1.22(0.83-1.80) | 0.318 |
|  |  | Recessive |  |  |  | 1.03(0.45-2.34) | 0.950 |  | 0.95(0.38-2.37) | 0.913 |
|  | rs2298881 | CC | 125(35.2) | 44 |  | ref. |  |  | ref. |  |
|  |  | AC | 177(49.9) | 46 |  | 0.72(0.48-1.09) | 0.122 |  | 0.78(0.51-1.18) | 0.236 |
|  |  | AA | 53(14.9) | 14 |  | 0.69(0.38-1.27) | 0.235 |  | 0.70(0.38-1.28) | 0.245 |
|  |  | Dominant |  |  |  | 0.71(0.48-1.05) | 0.090 |  | 0.76(0.51-1.12) | 0.166 |
|  |  | Recessive |  |  |  | 0.83(0.47-1.45) | 0.509 |  | 0.82(0.46-1.44) | 0.481 |
|  | rs3212955 | AA | 176(49.6) | 46 |  | ref. |  |  | ref. |  |
|  |  | AG | 145(40.8) | 45 |  | 1.22(0.81-1.85) | 0.338 |  | 1.38(0.90-2.11) | 0.136 |
|  |  | GG | 34(9.6) | 13 |  | 1.45(0.79-2.69) | 0.235 |  | 1.48(0.79-2.76) | 0.220 |
|  |  | Dominant |  |  |  | 1.27(0.86-1.87) | 0.232 |  | 1.38(0.93-2.05) | 0.107 |
|  |  | Recessive |  |  |  | 1.31(0.73-2.35) | 0.362 |  | 1.20(0.67-2.16) | 0.536 |
|  | rs3212961 | CC | 92(25.9) | 34 |  | ref. |  |  | ref. |  |
|  |  | AC | 189(53.2) | 53 |  | 0.73(0.47-1.12) | 0.149 |  | 0.67(0.43-1.04) | 0.076 |
|  |  | AA | 74(20.8) | 17 |  | **0.52(0.29-0.94)** | **0.031** |  | **0.54(0.30-0.99)** | **0.045** |
|  |  | Dominant |  |  |  | 0.66(0.44-1.00) | 0.050 |  | **0.63(0.41-0.95)** | **0.028** |
|  |  | Recessive |  |  |  | 0.63(0.37-1.08) | 0.093 |  | 0.70(0.41-1.19) | 0.185 |
|  | rs3212986 | GG | 162(43.4) | 45 |  | ref. |  |  | ref. |  |
|  |  | GT | 152(42.8) | 46 |  | 1.22(0.80-1.84) | 0.353 |  | 1.40(0.92-2.13) | 0.115 |
|  |  | TT | 41(11.5) | 13 |  | 1.12(0.61-2.09) | 0.712 |  | 1.23(0.66-2.31) | 0.518 |
|  |  | Dominant |  |  |  | 1.20(0.81-1.77) | 0.369 |  | 1.34(0.91-2.00) | 0.142 |
|  |  | Recessive |  |  |  | 1.01(0.57-1.81) | 0.971 |  | 1.00(0.55-1.79) | 0.988 |
|  | rs735482 | AA | 109(30.7) | 35 |  | ref. |  |  | ref. |  |
|  |  | AC | 183(51.5) | 52 |  | 0.83(0.54-1.28) | 0.402 |  | 0.75(0.48-1.16) | 0.197 |
|  |  | CC | 63(17.7) | 17 |  | 0.72(0.40-1.31) | 0.281 |  | 0.70(0.38-1.27) | 0.239 |
|  |  | Dominant |  |  |  | 0.80(0.53-1.21) | 0.288 |  | 0.73(0.48-1.10) | 0.135 |
|  |  | Recessive |  |  |  | 0.81(0.47-1.37) | 0.427 |  | 0.83(0.49-1.42) | 0.494 |
|  |  |  |  |  |  |  |  |  |  |  |
| ERCC2 | rs1052555 | CC | 308(86.8) | 91 |  | ref. |  |  | ref. |  |
|  |  | CT | 46(13.0) | 12 |  | 0.78(0.43-1.42) | 0.414 |  | 0.98(0.54-1.81) | 0.956 |
|  |  | TT | 1(0.3) | 1 |  | / | / |  | / | / |
|  | rs13181 | TT | 299(84.2) | 89 |  | ref. |  |  | ref. |  |
|  |  | GT | 54(15.2) | 14 |  | 0.79(0.45-1.40) | 0.421 |  | 0.93(0.53-1.64) | 0.805 |
|  |  | GG | 2(0.6) | 1 |  | / | / |  | / | / |
|  | rs238406 | GG | 101(28.5) | 28 |  | ref. |  |  | ref. |  |
|  |  | GT | 181(51.0) | 53 |  | 1.11(0.70-1.76) | 0.648 |  | 1.02(0.64-1.63) | 0.922 |
|  |  | TT | 73(20.6) | 23 |  | 1.20(0.69-2.09) | 0.514 |  | 1.02(0.58-1.78) | 0.954 |
|  |  | Dominant |  |  |  | 1.13(0.73-1.75) | 0.571 |  | 1.02(0.66-1.57) | 0.940 |
|  |  | Recessive |  |  |  | 1.12(0.70-1.77) | 0.646 |  | 0.99(0.62-1.58) | 0.975 |
|  | rs238417 | GG | 105(29.6) | 33 |  | ref. |  |  | ref. |  |
|  |  | CG | 173(48.7) | 46 |  | 0.86(0.55-1.34) | 0.496 |  | 0.86(0.54-1.35) | 0.508 |
|  |  | CC | 77(21.7) | 25 |  | 0.98(0.58-1.65) | 0.942 |  | 0.89(0.53-1.50) | 0.663 |
|  |  | Dominant |  |  |  | 0.90(0.59-1.36) | 0.612 |  | 0.94(0.60-1.46) | 0.766 |
|  |  | Recessive |  |  |  | 1.07(0.68-1.69) | 0.756 |  | 0.97(0.62-1.53) | 0.903 |
|  | rs50871 | TT | 125(35.2) | 38 |  | ref. |  |  | ref. |  |
|  |  | GT | 207(58.3) | 54 |  | 0.98(0.64-1.49) | 0.912 |  | 0.89(0.58-1.36) | 0.577 |
|  |  | GG | 23(6.5) | 12 |  | **2.54(1.31-4.90)** | **0.006** |  | 1.74(0.86-3.50) | 0.121 |
|  |  | Dominant |  |  |  | 1.10(0.73-1.64) | 0.659 |  | 0.97(0.64-1.46) | 0.876 |
|  |  | Recessive |  |  |  | **2.55(1.39-4.66)** | **0.002** |  | 1.82(0.98-3.38) | 0.059 |
|  | rs50872 | CC | 212(59.7) | 65 |  | ref. |  |  | ref. |  |
|  |  | CT | 129(36.3) | 34 |  | 0.85(0.56-1.29) | 0.431 |  | 0.90(0.59-1.39) | 0.640 |
|  |  | TT | 14(3.9) | 5 |  | 1.19(0.48-2.96) | 0.706 |  | 1.06(0.42-2.67) | 0.903 |
|  |  | Dominant |  |  |  | 0.88(0.59-1.31) | 0.527 |  | 0.94(0.62-1.41) | 0.750 |
|  |  | Recessive |  |  |  | 1.27(0.52-3.12) | 0.604 |  | 1.17(0.47-2.90) | 0.740 |
|  |  |  |  |  |  |  |  |  |  |  |
| ERCC3 | rs4150441 | GG | 122(32.7) | 35 |  | ref. |  |  | ref. |  |
|  |  | AG | 165(46.5) | 46 |  | 0.98(0.63-1.52) | 0.910 |  | 1.03(0.66-1.63) | 0.887 |
|  |  | AA | 68(19.2) | 23 |  | 1.38(0.82-2.34) | 0.230 |  | 1.35(0.79-2.31) | 0.269 |
|  |  | Dominant |  |  |  | 1.09(0.72-1.64) | 0.687 |  | 1.12(0.74-1.70) | 0.588 |
|  |  | Recessive |  |  |  | 1.43(0.90-2.27) | 0.133 |  | 1.38(0.86-2.20) | 0.178 |
|  | rs4150448 | GG | 285(80.3) | 83 |  | ref. |  |  | ref. |  |
|  |  | AG | 62(17.5) | 20 |  | 1.25(0.76-2.05) | 0.388 |  | 1.40(0.85-2.32) | 0.186 |
|  |  | AA | 8(2.3) | 1 |  | 0.37(0.05-2.66) | 0.323 |  | 0.31(0.04-2.25) | 0.247 |
|  |  | Dominant |  |  |  | 1.11(0.68-1.81) | 0.672 |  | 1.19(0.73-1.94) | 0.489 |
|  |  | Recessive |  |  |  | 0.35(0.05-2.53) | 0.300 |  | 0.29(0.04-2.11) | 0.223 |
|  | rs4150506 | CC | 175(49.3) | 50 |  | ref. |  |  | ref. |  |
|  |  | CT | 142(40.0) | 43 |  | 1.07(0.71-1.61) | 0.758 |  | 1.25(0.82-1.90) | 0.293 |
|  |  | TT | 38(10.7) | 11 |  | 1.13(0.59-2.17) | 0.715 |  | 0.98(0.51-1.90) | 0.961 |
|  |  | Dominant |  |  |  | 1.08(0.73-1.59) | 0.695 |  | 1.18(0.80-1.75) | 0.408 |
|  |  | Recessive |  |  |  | 1.10(0.59-2.05) | 0.772 |  | 0.90(0.48-1.68) | 0.731 |
|  |  |  |  |  |  |  |  |  |  |  |
| ERCC4 | rs6498486 | AA | 126(60.3) | 49 |  | ref. |  |  | ref. |  |
|  |  | AC | 72(34.4) | 28 |  | 1.00(0.63-1.59) | 0.999 |  | 0.92(0.57-1.47) | 0.720 |
|  |  | CC | 11(5.3) | 4 |  | 1.01(0.36-2.79) | 0.991 |  | 0.74(0.27-2.06) | 0.566 |
|  |  | Dominant |  |  |  | 1.00(0.64-1.56) | 0.997 |  | 0.89(0.57-1.40) | 0.624 |
|  |  | Recessive |  |  |  | 0.99(0.36-2.70) | 0.979 |  | 0.74(0.27-2.03) | 0.560 |
|  | rs1799801 | TT | 223(62.8) | 63 |  | ref. |  |  | ref. |  |
|  |  | CT | 115(32.4) | 33 |  | 1.02(0.67-1.56) | 0.921 |  | 0.98(0.64-1.51) | 0.930 |
|  |  | CC | 17(4.8) | 8 |  | 1.78(0.85-3.71) | 0.126 |  | 1.11(0.53-2.36) | 0.782 |
|  |  | Dominant |  |  |  | 1.12(0.75-1.66) | 0.590 |  | 1.00(0.67-1.48) | 0.967 |
|  |  | Recessive |  |  |  | 1.76(0.86-3.63) | 0.124 |  | 1.07(0.51-2.23) | 0.857 |
|  | rs2276464 | GG | 225(63.4) | 64 |  | ref. |  |  | ref. |  |
|  |  | CG | 113(31.8) | 32 |  | 1.01(0.65-1.54) | 0.982 |  | 0.95(0.62-1.47) | 0.829 |
|  |  | CC | 17(4.8) | 8 |  | 1.76(0.85-3.68) | 0.131 |  | 1.10(0.52-2.33) | 0.804 |
|  |  | Dominant |  |  |  | 1.10(0.74-1.64) | 0.636 |  | 0.97(0.65-1.45) | 0.878 |
|  |  | Recessive |  |  |  | 1.76(0.86-3.63) | 0.124 |  | 1.07(0.51-2.23) | 0.857 |
|  | rs254942 | TT | 233(63.0) | 70 |  | ref. |  |  | ref. |  |
|  |  | CT | 115(31.1) | 32 |  | 1.02(0.67-1.55) | 0.927 |  | 1.15(0.75-1.76) | 0.534 |
|  |  | CC | 22(5.9) | 9 |  | 1.27(0.63-2.54) | 0.501 |  | 0.96(0.47-1.93) | 0.898 |
|  |  | Dominant |  |  |  | 1.06(0.72-1.57) | 0.752 |  | 1.12(0.75-1.65) | 0.588 |
|  |  | Recessive |  |  |  | 1.26(0.64-2.49) | 0.506 |  | 0.94(0.47-1.86) | 0.853 |
|  |  |  |  |  |  |  |  |  |  |  |
| ERCC5 | rs1047768 | TT | 163(46.2) | 48 |  | ref. |  |  | ref. |  |
|  |  | CT | 160(45.3) | 45 |  | 0.99(0.66-1.49) | 0.971 |  | 1.04(0.68-1.58) | 0.865 |
|  |  | CC | 30(8.5) | 10 |  | 1.15(0.58-2.28) | 0.682 |  | 1.29(0.64-2.63) | 0.478 |
|  |  | Dominant |  |  |  | 1.02(0.69-1.51) | 0.909 |  | 1.09(0.73-1.63) | 0.666 |
|  |  | Recessive |  |  |  | 1.16(0.61-2.24) | 0.649 |  | 1.36(0.69-2.67) | 0.376 |
|  | rs2094258 | GG | 149(42.1) | 53 |  | ref. |  |  | ref. |  |
|  |  | AG | 162(45.8) | 39 |  | **0.59(0.39-0.90)** | **0.014** |  | 0.67(0.44-1.03) | 0.068 |
|  |  | AA | 43(12.1) | 12 |  | 0.68(0.36-1.28) | 0.231 |  | 0.69(0.36-1.32) | 0.262 |
|  |  | Dominant |  |  |  | **0.61(0.42-0.90)** | **0.013** |  | **0.65(0.44-0.97)** | **0.033** |
|  |  | Recessive |  |  |  | 0.88(0.48-1.61) | 0.683 |  | 0.76(0.41-1.42) | 0.391 |
|  | rs2298959 | CC | 324(91.3) | 94 |  | ref. |  |  | ref. |  |
|  |  | AC | 29(8.2) | 9 |  | 1.18(0.60-2.35) | 0.630 |  | 0.88(0.43-1.82) | 0.736 |
|  |  | AA | 2(0.6) | 1 |  | / | / |  | / | / |
|  | rs2296147 | TT | 223(60.4) | 68 |  | ref. |  |  | ref. |  |
|  |  | CT | 131(35.5) | 38 |  | 1.00(0.67-1.49) | 0.986 |  | 1.03(0.68-1.54) | 0.896 |
|  |  | CC | 15(4.1) | 5 |  | 1.14(0.46-2.84) | 0.773 |  | 1.40(0.56-3.51) | 0.469 |
|  |  | Dominant |  |  |  | 1.01(0.69-1.49) | 0.948 |  | 1.06(0.71-1.56) | 0.783 |
|  |  | Recessive |  |  |  | 1.13(0.46-2.77) | 0.790 |  | 1.32(0.53-3.25) | 0.550 |
|  | rs4150291 | AA | 288(81.1) | 88 |  | ref. |  |  | ref. |  |
|  |  | AT | 63(17.7) | 16 |  | 0.76(0.45-1.30) | 0.312 |  | 0.76(0.44-1.30) | 0.317 |
|  |  | TT | 4(1.1) | 0 |  | / | / |  | / | / |
|  | rs4150383 | GG | 311(87.6) | 95 |  | ref. |  |  | ref. |  |
|  |  | AG | 43(12.1) | 9 |  | 1.53(0.77-3.03) | 0.222 |  | 0.58(0.29-1.16) | 0.126 |
|  |  | AA | 1(0.3) | 0 |  | / | / |  | / | / |
|  | rs751402 | CC | 144(41.1) | 37 |  | ref. |  |  | ref. |  |
|  |  | CT | 161(46.0) | 53 |  | 1.26(0.83-1.93) | 0.280 |  | 1.17(0.77-1.80) | 0.464 |
|  |  | TT | 45(12.9) | 16 |  | 1.49(0.83-2.69) | 0.184 |  | 1.50(0.81-2.77) | 0.196 |
|  |  | Dominant |  |  |  | 1.31(0.88-1.96) | 0.188 |  | 1.25(0.84-1.88) | 0.275 |
|  |  | Recessive |  |  |  | 1.32(0.77-2.24) | 0.313 |  | 1.44(0.83-2.50) | 0.195 |
|  | rs873601 | GG | 95(26.8) | 31 |  | ref. |  |  | ref. |  |
|  |  | AG | 180(50.7) | 46 |  | 0.77(0.49-1.22) | 0.272 |  | 0.74(0.45-1.20) | 0.217 |
|  |  | AA | 80(22.5) | 27 |  | 1.23(0.73-2.06) | 0.439 |  | 1.10(0.64-1.89) | 0.735 |
|  |  | Dominant |  |  |  | 0.90(0.59-1.37) | 0.608 |  | 0.85(0.55-1.32) | 0.467 |
|  |  | Recessive |  |  |  | 1.44(0.93-2.23) | 0.106 |  | 1.28(0.81-2.03) | 0.297 |
|  |  |  |  |  |  |  |  |  |  |  |
| ERCC6 | rs1917799 | TT | 70(32.6) | 22 |  | ref. |  |  | ref. |  |
|  |  | GT | 105(48.8) | 45 |  | 1.44(0.86-2.40) | 0.162 |  | **1.68(1.01-2.81)** | **0.048** |
|  |  | GG | 40(18.6) | 15 |  | 1.23(0.64-2.37) | 0.540 |  | 0.95(0.48-1.88) | 0.874 |
|  |  | Dominant |  |  |  | 1.40(0.85-2.26) | 0.191 |  | 1.47(0.90-2.41) | 0.124 |
|  |  | Recessive |  |  |  | 0.98(0.56-1.72) | 0.944 |  | 0.78(0.44-1.38) | 0.400 |
|  |  |  |  |  |  |  |  |  |  |  |
| ERCC8 | rs158572 | AA | 173(80.5) | 68 |  | ref. |  |  | ref. |  |
|  |  | AG | 38(17.7) | 13 |  | 0.86(0.48-1.56) | 0.623 |  | 0.84(0.46-1.54) | 0.564 |
|  |  | GG | 4(1.9) | 1 |  | 0.62(0.09-4.44) | 0.631 |  | 0.90(0.12-6.58) | 0.914 |
|  | rs158916 | TT | 167(77.7) | 66 |  | ref. |  |  | ref. |  |
|  |  | CT | 44(20.5) | 15 |  | 0.84(0.48-1.47) | 0.531 |  | 0.72(0.41-1.27) | 0.250 |
|  |  | CC | 4(1.9) | 1 |  | 0.68(0.09-4.91) | 0.703 |  | 0.64(0.09-4.70) | 0.661 |
|  |  |  |  |  |  |  |  |  |  |  |
| XPA | rs10817938 | TT | 207(58.5) | 56 |  | ref. |  |  | ref. |  |
|  |  | CT | 127(35.9) | 42 |  | 1.18(0.79-1.76) | 0.424 |  | 1.12(0.74-1.68) | 0.602 |
|  |  | CC | 20(5.6) | 6 |  | 0.99(0.43-2.30) | 0.978 |  | 1.23(0.53-2.87) | 0.638 |
|  |  | Dominant |  |  |  | 1.15(0.78-1.70) | 0.480 |  | 1.14(0.77-1.69) | 0.516 |
|  |  | Recessive |  |  |  | 0.93(0.41-2.12) | 0.861 |  | 1.25(0.55-2.87) | 0.592 |
|  | rs2808668 | TT | 86(24.3) | 24 |  | ref. |  |  | ref. |  |
|  |  | CT | 183(51.7) | 58 |  | 1.22(0.75-1.97) | 0.424 |  | 1.03(0.63-1.68) | 0.910 |
|  |  | CC | 85(24.0) | 22 |  | 1.01(0.56-1.81) | 0.981 |  | 0.87(0.48-1.59) | 0.651 |
|  |  | Dominant |  |  |  | 1.15(0.73-1.83) | 0.549 |  | 1.00(0.62-1.59) | 0.990 |
|  |  | Recessive |  |  |  | 0.87(0.54-1.40) | 0.570 |  | 0.86(0.53-1.39) | 0.537 |
|  | rs3176629 | CC | 289(81.4) | 86 |  | ref. |  |  | ref. |  |
|  |  | CT | 64(18.0) | 17 |  | 0.90(0.54-1.52) | 0.703 |  | 0.99(0.58-1.67) | 0.957 |
|  |  | TT | 2(0.6) | 1 |  | / | / |  | / | / |
|  |  |  |  |  |  |  |  |  |  |  |
| XPC | rs1870134 | GG | 185(52.1) | 50 |  | ref. |  |  | ref. |  |
|  |  | CG | 149(42.0) | 46 |  | 1.21(0.81-1.82) | 0.345 |  | 1.06(0.70-1.59) | 0.786 |
|  |  | CC | 21(5.9) | 8 |  | 1.59(0.75-3.36) | 0.226 |  | 1.16(0.53-2.53) | 0.711 |
|  |  | Dominant |  |  |  | 1.26(0.86-1.86) | 0.238 |  | 1.07(0.72-1.59) | 0.733 |
|  |  | Recessive |  |  |  | 1.47(0.71-3.03) | 0.297 |  | 1.13(0.54-2.36) | 0.746 |
|  | rs2228000 | CC | 171(45.8) | 47 |  | ref. |  |  | ref. |  |
|  |  | CT | 145(41.0) | 48 |  | 1.24(0.83-1.86) | 0.293 |  | 1.31(0.87-1.98) | 0.199 |
|  |  | TT | 38(10.7) | 9 |  | 0.79(0.39-1.61) | 0.510 |  | 0.88(0.43-1.81) | 0.729 |
|  |  | Dominant |  |  |  | 1.14(0.77-1.68) | 0.513 |  | 1.21(0.82-1.80) | 0.337 |
|  |  | Recessive |  |  |  | 0.71(0.36-1.41) | 0.326 |  | 0.76(0.38-1.51) | 0.435 |
|  | rs2228001 | AA | 138(38.9) | 46 |  | ref. |  |  | ref. |  |
|  |  | AC | 168(47.3) | 44 |  | 0.73(0.48-1.11) | 0.143 |  | 0.81(0.53-1.24) | 0.340 |
|  |  | CC | 49(13.8) | 14 |  | 0.84(0.46-1.52) | 0.555 |  | 1.06(0.58-1.93) | 0.861 |
|  |  | Dominant |  |  |  | 0.76(0.52-1.12) | 0.169 |  | 0.87(0.58-1.28) | 0.469 |
|  |  | Recessive |  |  |  | 1.00(0.57-1.75) | 0.989 |  | 1.15(0.65-2.03) | 0.628 |
|  | rs2470352 | AA | 350(98.6) | 104 |  | ref. |  |  | ref. |  |
|  |  | AT | 5(1.4) | 0 |  | / | / |  | / | / |
|  | rs2607775 | CC | 320 | 96 |  | ref. |  |  | ref. |  |
|  |  | CG | 35 | 8 |  | 0.73(0.36-1.50) | 0.393 |  | 0.67(0.33-1.39) | 0.287 |
|  |  |  |  |  |  |  |  |  |  |  |
| DDB2 | rs2029298 | GG | 159(44.8) | 49 |  | ref. |  |  | ref. |  |
|  |  | AG | 162(45.6) | 42 |  | 0.80(0.53-1.21) | 0.294 |  | 0.83(0.54-1.28) | 0.398 |
|  |  | AA | 34(9.6) | 13 |  | 1.35(0.73-2.49) | 0.343 |  | 1.29(0.69-2.41) | 0.418 |
|  |  | Dominant |  |  |  | 0.89(0.60-1.31) | 0.542 |  | 0.91(0.61-1.35) | 0.626 |
|  |  | Recessive |  |  |  | 1.50(0.84-2.68) | 0.173 |  | 1.43(0.80-2.57) | 0.229 |
|  | rs326222 | TT | 180(50.7) | 47 |  | ref. |  |  | ref. |  |
|  |  | CT | 160(45.1) | 52 |  | 1.23(0.83-1.84) | 0.299 |  | 1.39(0.92-2.11) | 0.121 |
|  |  | CC | 15(4.2) | 5 |  | 1.62(0.64-4.10) | 0.305 |  | 2.23(0.86-5.79) | 0.099 |
|  |  | Dominant |  |  |  | 1.27(0.86-1.87) | 0.236 |  | 1.43(0.95-2.15) | 0.086 |
|  |  | Recessive |  |  |  | 1.50(0.61-3.70) | 0.376 |  | 2.05(0.82-14.85) | 0.124 |
|  | rs3781619 | AA | 132(37.2) | 34 |  | ref. |  |  | ref. |  |
|  |  | AG | 187(52.7) | 54 |  | 1.18(0.76-1.82) | 0.456 |  | 1.20(0.77-1.88) | 0.419 |
|  |  | GG | 36(10.1) | 16 |  | **2.06(1.13-3.75)** | **0.018** |  | **2.40(1.27-4.55)** | **0.007** |
|  |  | Dominant |  |  |  | 1.31(0.87-1.98) | 0.201 |  | 1.37(0.90-2.10) | 0.146 |
|  |  | Recessive |  |  |  | **1.89(1.11-3.22)** | **0.019** |  | **2.30(1.33-3.97)** | **0.003** |
|  | rs830083 | CC | 102(28.8) | 32 |  | ref. |  |  | ref. |  |
|  |  | CG | 163(46.0) | 45 |  | 0.66(0.41-1.04) | 0.073 |  | **0.61(0.38-0.98)** | **0.042** |
|  |  | GG | 89(25.1) | 27 |  | 0.77(0.46-1.30) | 0.324 |  | 0.81(0.48-1.38) | 0.442 |
|  |  | Dominant |  |  |  | 0.70(0.46-1.06) | 0.093 |  | 0.66(0.43-1.01) | 0.056 |
|  |  | Recessive |  |  |  | 0.91(0.58-1.41) | 0.657 |  | 0.99(0.63-1.56) | 0.957 |

a, Calculated by Cox proportional model using univariate analysis.

b, Calculated by Cox proportional model using multivariate analysis.
